# Full arch fixed prostheses vs. full arch telescopic-retained retrievable prostheses both supported by implants and natural tooth abutments in periodontally treated patients: Results at 15 years

**DOI:** 10.4317/jced.55904

**Published:** 2019-10-01

**Authors:** Renzo Guarnieri, Dario Di Nardo, Gianni Di Giorgio, Gabriele Miccoli, Luca Testarelli

**Affiliations:** 1 MD, DDS. Dept. of Dental and Maxillofacial Sciences, School of Dentistry, University La Sapienza, Rome, Italy; 2 DDS, PhD. Dept. of Dental and Maxillofacial Sciences, School of Dentistry, University La Sapienza, Rome, Italy

## Abstract

**Background:**

The clinical outcome of full arch fixed prostheses vs. full arch telescopic-retained retrievable prostheses supported by implants and natural tooth abutments in periodontally treated patients has been reported by few studies, with controversial results. The objective of this study was to evaluate long-term (15 years) complications of abutment teeth and dental implants in periodontally treated patients, rehabilitated with full arch telescopic-retained retrievable prostheses (TRP)s vs. full arch fixed prostheses (FP)s supported by teeth and implants.

**Material and Methods:**

After active periodontal therapy (non-surgical and surgical), and implant placement (replacement of hopeless teeth and in edentulous sites), 18 patients were rehabilitated in both dental arches with full arch TRPs, and 17 patients were rehabilitated with full arch FPs. Patients were annually recalled for technical and/or biological complications monitoring.

**Results:**

During the 15-year observation period, 29 of 164 (17.6%) implants failed in the TRP group and 26 of 152 (17.1 %) implants in the FP group. Due to progression of periodontal disease, endo-perio untreatable lesion and caries, 22 of 233 abutment teeth were extracted (8.1) % in the TRP group and 23 of 221 (10.4%) abutment teeth were extracted in the FP group. Difference in implant failures and abutment teeth loss between the two groups were found not statistically significant (*p* >0.05). Poisson regression analysis showed that in both groups, factors such as smoking habits, FMBS>20, number of pockets >6 mm, mean bone loss, and bone loss/age, contribute to tooth and implant failure (*p*<0.05).

**Conclusions:**

In this clinical study, in periodontally treated patients, full arch telescopic-retained retrievable prostheses, and full arch fixed prosthesis, supported by teeth and implants presented comparable long-term results of tooth loss and implant failure, if regular periodontal therapy is implemented.

** Key words:**Periodontal disease, implants, tooth-implant connection, telescopic prosthesis, fixed prosthesis.

## Introduction

Dentitions, damaged by severe periodontal disease, need comprehensive treatment plans encompassing non-surgical and surgical periodontal therapies, as well as prosthetic rehabilitation, to restore health, function and aesthetics (1-3). Over the last several decades, the use of osseointegrated implants has shown long-term high percentages of success in both healthy and periodontally compromised partially edentulous subjects (4,5). Since the prognosis of complex periodontal therapy may not match the high levels of success of treatment with implants, some authors suggested that teeth with a predicted hopeless/questionable prognosis should not be treated periodontally but replaced with implants (6,7). However, recent literature data indicate that dental implants in periodontally compromised patients could yield lower survival rates and higher mean marginal bone loss (MBL) compared with those of implants placed in periodontally healthy subjects (8,9). Moreover, an increased incidence of peri-implantitis was observed in periodontally compromised patients compared to healthy patients (10). Due to implications of additional cost and patient morbidity associated with implant placement, other authors suggested to retain teeth as long as possible, whilst performing active and supportive periodontal treatment, and to only replace teeth later on if absolutely necessary (11). Nevertheless, because full masticatory function requires a sufficient number of occluding posterior units to avoid cantilever extensions (12) the use of supplemental, so-called strategic, implants could be recommended to enhance the clinical performance when a reduced number of abutment teeth are present.

Contrasting data are present in literature on the use of combined tooth-implant supported restorations (13-18). Due to biologic and prosthetic reasons, some authors suggested to avoid connection between rigid ankylosed implants and relatively mobile dentition. In contrast, other authors explained that there is sufficient flexibility within the implant restoration unit to allow for movement of the tooth within the socket to a degree where support is also achieved from the tooth (15,18).

There are just few trials assessing in periodontally treated and maintained patients the function of restorations supported by tooth-implant combination according to the type of prosthodontic treatment. Therefore, the first objective of the current study was to assess the long-term rate of abutment tooth loss, and implant failure. The second objective is to compare outcomes of full arch telescopic-retained retrievable prosthesis (TRP) and full arch fixed prosthesis (FP), both supported by teeth-implants, used to rehabilitate periodontally compromised dentitions after active periodontal therapy (ATP).

## Material and Methods

This prospective clinical study comprised periodontally compromised patients treated and maintained with supportive periodontal therapy (SPT), who received full arch TRPs or full arch FPs, both supported by teeth-implants. The patient population comprised 35 periodontally compromised patients (17 women and 18 men) with a mean age of 49.7 years (±4.8) who were selected in one Italian private dental clinic to receive, during or after ATP, supplementary implants. In each patient, at least eight abutments (teeth plus implants) per arch were obtained for the prosthetic rehabilitation. Since all patients were previously treated either as part of an approved research protocol or as part of routine care using accepted therapy for each patient’s specific clinical needs, the study was granted an exemption by local institutional review board. All patients signed written informed consent forms. The declaration of Helsinki for human clinical trial was followed.

Patients were divided into two groups: 18 patients in TRP group, and 17 in FP group. In the TRP group, a total of 164 implants, (104 BioLok® Implants, now marketed by BioHorizons, Birmingham, AL, USA, and 60 P1H (P1H s.r.l. Implants, Villanova di S. Daniele, UD, Italy), were placed in addition to 233 teeth. For each arch, all natural teeth in combination with the strategic implants were provided with telescopic crowns. In the FP group, a total of 152 implants (91 BioLok® Implants, now marketed by BioHorizons, Birmingham, AL, USA, and 61 P1H (P1H s.r.l. Implant, Villanova di S. Daniele, UD, Italy), were placed in addition to 151 teeth. For each arch, all natural teeth in combination with the strategic implants were provided with fixed bridges.

Inclusion criteria: To be included in the study, each subject had to present at the first visit an inter-proximal attachment loss >3 mm and/or radiographic bone loss >30% of root length in >30% of sites. Moreover, patients must have been treated with non-surgical periodontal therapy followed or not by subsequent periodontal surgical therapy, and by implant therapy replacing hopeless teeth or restoring edentulous sites.

Exclusion criteria: Exclusion criteria included full mouth plaque score (FMPS) and full mouth bleeding score (FMBS) >25%, alcohol and drug abuse; pregnancy; uncontrolled systemic/metabolic disorders, no interest in participating in the study.

Pre-treatment clinical examination.

Gender, date of birth, smoking habits, medical history and treatment plan were obtained at the time of initial visit. Full mouth plaque score (FMPS), full mouth bleeding score (FMBS), pocket depth (PD), number of sites with plaque (P), and number of sites with bleeding on probing (BOP) were measured at the first clinical examination and the end of non-surgical therapeutic phase.

Tooth mobility according to Miller’s classification was evaluated. Moreover, subjects were radiographically monitored at baseline and at the end of the follow-up period.

Radiographs: Prior to APT and 15 years later, complete set of periapical radiographs using film holders were obtained for each patient. All radiographs were viewed in a darkened room using a radiograph screen. Relative bone loss in percent was assessed at the periodontally most affected site of each tooth using a Schei ruler to the nearest 10% (18). All radiographic assessments were performed by one examiner (RG). Mean of all relative bone loss assessments measured per patient were calculated to characterize an individual. For peri-implant marginal bone evaluation, an individualized acrylic resin device was used for initial and subsequent radiographs. A computer-assisted measurement automatically provided by a software program (VixWin Platinum Imaging Software; Gendex, Des Plaines, IL, USA) was used for radiographic measurements. The peri-implant radiographic marginal bone loss was calculated by subtracting the marginal bone level at baseline from marginal bone level at the last examination. In 5 patients, radiographic assessments were repeated after 14 days to assess reproducibility. In 94% of the cases, the difference between repeated measurements was ≤10%.

Tooth prognosis: Tooth prognosis was assigned to all teeth with available clinical and radiographic data. In the absence of a universally validated objective method for assigning tooth prognosis, the following variables were used: teeth with bone loss greater than 75% or teeth that had at least two characteristics of ‘questionable’ category, were classified as “hopeless”; teeth with bone loss between 50% and 75%, or the presence of an angular defect or furcation involvement were considered “questionable”; teeth with less than 50% bone loss or not fitting one of the two previous categories were considered “good”. Angular defects in interproximal areas were recorded when they extended more than 2mm from the existing bone level to the depth of the defect or extended more than half the root length.

Evaluation of patients’ charts: The following information was retrieved from the patients’ charts:

1. Tooth loss and implant failure after APT were assessed by comparison of the first and last (≥15 years later) examinations.

2. Each patient was assigned a baseline diagnosis (e.g. aggressive/chronic periodontitis) according to the actual classification of periodontal diseases.

3. According to their self-reported smoking history, patients were categorized as current, or former smokers, or as non-smokers. Non-smokers were patients who had never smoked in their lives and patients who had quit smoking at least 5 years prior to treatment. All other patients were classified as current or former smokers.

Active Periodontal Therapy (APT) included appropriate initial therapy consisting in motivation, oral hygiene instruction and scaling and root planning, with the aim to reduce to a minimal level periodontal pathogens, followed by, when indicated, additional periodontal and implant surgery (including resective, regenerative or muco-gingival plastic surgery) and conservative, endodontic and prosthetic treatment if necessary. No adjunctive therapy consisting of systemic or local antibiotics during APT was used. According to the initial treatment plan, teeth which were considered to have hopeless prognosis were extracted during APT. Following initial therapy, if PPD was ≥5 mm with signs of inflammation like bleeding on probing (BOP), additional access flap surgery was performed. Pocket elimination surgery, osseous resection, or augmentative regenerative procedures were attempted, if needed. Furcation involvement (FI) was diagnosed clinically by probing (Nabers probe) and classified according to Hamp et al (19). For teeth with advanced FI (degrees II and III), furcation debridement was performed with diamond-coated sonic-scaler inserts during flap surgery .

Implant placement: Dental implants were placed, under local anesthesia, according to the manufacturer’s instructions during or after the periodontal surgery. All implants were placed using a standardized two-stage surgical procedure, with the border of the rough surface approximating the alveolar bone crest leaving the machined neck portion in the transmucosal area. Implants in extraction sites that required extensive bone augmentation were inserted 3 months after extractions. If necessary, an excision of soft tissue was performed in order to allow a close adaptation of the wound margins, submerging the implant. The number and position of implants in each patient were determined after a thorough diagnosis of the anticipated needs for the planned prosthesis and the presence of anatomic limitations. Appropriate healing screws were placed 4-6 months after implant installation, and abutment connection was carried out 3-6 months post-surgery.

Prosthodontic procedure:

Small preparation angles, and almost parallel preparations of the abutment teeth were preferred to create a secure retention. The presence of long clinical crowns, as a sequel of loss of clinical attachment and/or pocket elimination periodontal surgery, was favorable in terms of retention and resistance form. After final teeth preparation, impressions were obtained using screw-retained implant impression copings and customized trays with polyether material (Impregum 3 M Espe, Seefeld, Austria). During the same appointment, a preliminary bite registration was taken. Master models were fabricated using die stone with implant analogues and a flexible gingival mask. Prosthetic margins were located supragingival.

TRP group: On the stone models, implant abutments and prepared teeth were waxed up using dome shaped primary copings and casted with cobalt–chromium alloy. Implant abutments were individualized based on length and parallelism. The castings were milled using a paralleling cutting device. The primary copings for the prepared teeth were designed with a wall thickness of 0.1– 0.2 mm. A putty impression with the primary coping in place was made for the fabrication of the framework. The framework with secondary copings were waxed up on the refractory cast and casted in cobalt chromium alloy. The frameworks were veneered using a conventional built-up technique. The veneering porcelain (Esprident Triceram; Dentaurum, Ispringen, Germany) was fired in layers on the framework. The copings were permanently cemented onto the prepared teeth with glass-ionomer cement (Fuji I, GC, Tokyo, Japan), and screwed on the implants. Retention of the denture was finally provided by the friction between the parallel surfaces of the primary and secondary copings.

FP group: The framework was constructed using Wirobond® C (Bego, Bremen, Germany), a nickel-free cobalt/chromium (Co/Cr) alloy with veneering capacity. Metal try-in/jaw registration and porcelain try-in at bisque bake stage allowed refining of the occlusion. The veneering porcelain (Esprident Triceram; Dentaurum, Ispringen, Germany) was fired in layers on the framework. The final bridge was cemented semi- permanently using Rely X Temp NE (3M Espe).

Follow-up.

Patients were recalled at various intervals (from 3 to six months), depending on the initial diagnosis and the results of the therapy, for SPT. Motivation, reinstruction, instrumentation and treatment of re-infected sites were performed as needed. Patients were placed on an individually tailored maintenance care program, including continuous evaluation of the occurrence and the risk of disease progression.

Supportive periodontal therapy (SPT): Supportive periodontal therapy encompassed the following elements for all patients at each appointment: Assessment of approximal plaque index, re-instruction and re-motivation to achieve effective individual plaque control, professional tooth cleaning with hand instruments and polishing of all teeth using rubber cups and polishing paste and application of a fluoride gel. A dental status and PPD were obtained at 6 sites per tooth on a semi-annual basis. Thirty seconds after probing, BOP was recorded. Sites exhibiting PPD = 4 mm and BOP as well as sites with PPD ≥5 mm were scaled subgingivally.

Data assessment and analyses.

The following criteria were adopted to evaluate the clinical success of APT: no PPD ≥4 mm, minimal BOP (<25%), low plaque index (<30%), aesthetically satisfactory periodontal situation, absence of pain, satisfactory function. Bone loss at 1st year <1.5 mm, annual bone loss <0.2 mm thereafter, absence of mobility, pain, infection, and radiolucent area around the implant were considered parameters of implant success. Radiographic proximal bone loss of at least three threads when compared with bone levels 1 year after loading associated to BOP and suppuration, were considered parameters for diagnosis of peri-implantitis.

Complication-related data were assessed from the patient records and included biological complications (progression of periodontal disease, implant failure, tooth extraction), restorative complications (caries, root canal infection), and prosthetic complications (de-cementation of crowns, unscrewing of abutment, screw or abutment fracture, restoration or veneering porcelain fracture, implant or tooth fracture, intrusion of abutment teeth, restoration replacement).

Statistical analyses were performed with IBM SPSS Statistics version 19 (IBM Corp). The observation period started with the day of prosthesis insertion and ended with the day of clinical reevaluation after 15 years. Data was analysed descriptively (mean and standard deviations), and groups compared using independent-samples t-test. Statistical significance level was set at *p* < 0.05. Poisson regression was used in each group to test predictors (sex, patient age, location (maxilla and/or mandible), periodontal diagnosis, smoking habit, FMBS>20, number of pockets >6 mm, mean bone loss and bone loss/age) and their possible effect on success. *P*<0.05 was regarded as indicative of exploratory significant difference.

## Results

In  Table 1 are reported details of patients, teeth, abutment teeth loss, and implant failures, for TRP and FP groups, before APT (T0), at the beginning of follow-up (T1) and end of follow-up (T2). In  Table 2 are reported the positions of implants according to treatment groups at T1 and T2.

**Table 1:**
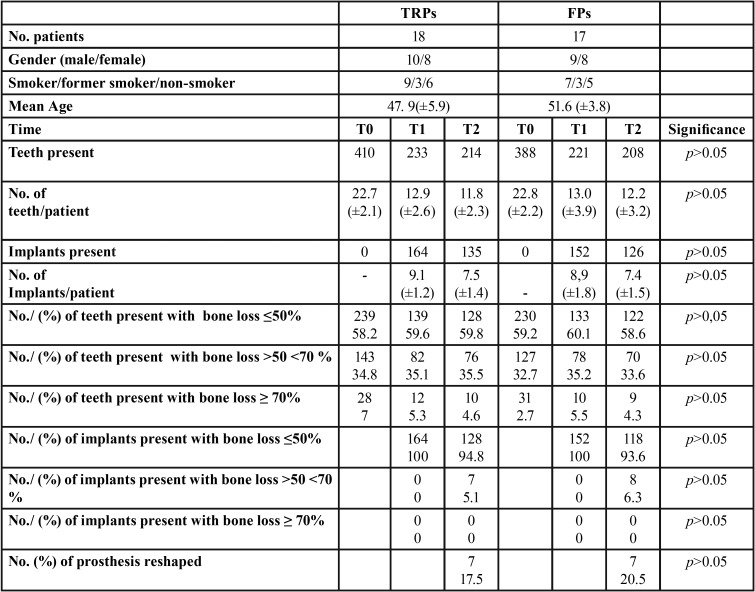
Details of patients, teeth, abutment teeth loss, and implant failures, for TRPs group and FPs group, before APT (T0) and at the beginning (T1) and end (T2) of follow-up.

**Table 2:**
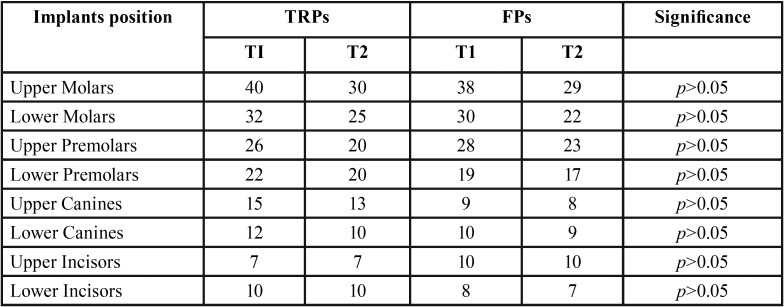
Positions of implants according to treatment groups at the beginning (T1) and end (T2) of follow-up.

SEVER COMPLICATIONS:

TRP group: During the 15-year observation period, 29 implant failures occurred after the delivery of TRP (82.4 % implant survival rate). Five, 7, 7, 5, and 5 implants were removed after 6,7,8,9, and 13 years, respectively. Twenty-seven implants were removed for peri-implantitis. In addition, 11 teeth were extracted after prosthetic treatment due to progression of the periodontal disease (2, 2, 3, 2, and 2 teeth after 4, 5, 7, 9 and 12 years respectively). Furthermore, 7 teeth were extracted after 6, 7, 7, 9, 10, 11, and 12 years, respectively, due to endo-perio untreatable lesion and 4 teeth, after 4, 5, 7, and 9 years, respectively, due to caries (90.6 % tooth survival rate). The restorations were reshaped and thereafter positioned again. Thus, seven TRP had to be subsequently changed (87.7 % unchanged TRPs after 15 years; but all 36 complete-arch restorations remained functional (100 % TRPs survival rate).

FP group: During the 15-year observation period, 26 implant failures occurred after the delivery of FDPs (82.9. % implant survival rate). Seven, 6, 6, 4, and 3 implants were removed after 6, 6, 8, 9 and 13 years, respectively. Twenty-three implants were removed for peri-implantitis. In addition, 23 abutment teeth were extracted after prosthetic treatment (10.4%). Eleven teeth were removed due to progression of the periodontal disease (3 teeth after 8 years, 2 teeth after 10 years, 4 teeth after 13 years, and 2 teeth after 14 years). Eight teeth were extracted after 5, 6, 7, 7, 9, 10, 11, and 12 years, respectively, due to endo-perio untreatable lesions. Additionally, 2 teeth were removed after 6, and 8 years, and 2 teeth were removed after 7, and 12 years, respectively, due to caries and root fractures (89.6 % tooth survival rate). The restorations were reshaped and thereafter positioned again. Thus, 7 FDPs had to be subsequently changed (82.5 % unchanged FDPs after 15 years). Between two groups, no significant statistical difference of tooth loss and implant failure was found (*p* > 0.05). Furthermore, no statistical differences were found for implant failures between two groups in relation to the implant positions (*p* > 0.05, t-test) ( Table 2). Poisson regression ( Table 3) showed no statistically significant correlation between tooth loss/implant failures (*p* > 0.05) and sex, patient age and location (maxilla and/or mandible). A statistically significant correlation was noted between periodontal diagnosis, smoking habit, FMBS>20, number of pockets >6 mm, mean bone loss, and bone loss/age ( Table 4).

**Table 3:**
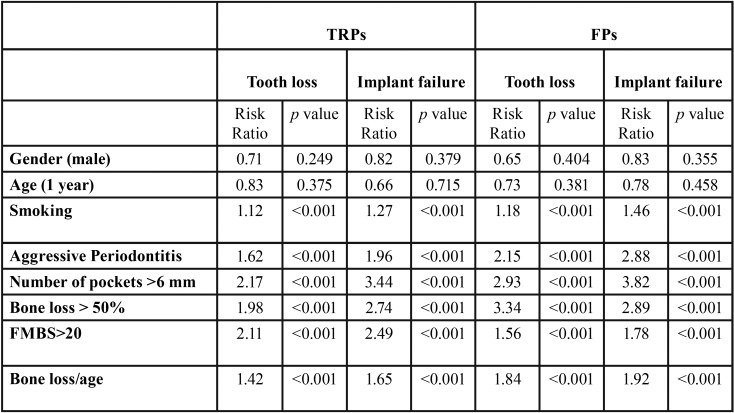
Abutment tooth loss and implant failure during the 15-year follow-up (Poisson regression analysis).

**Table 4:**
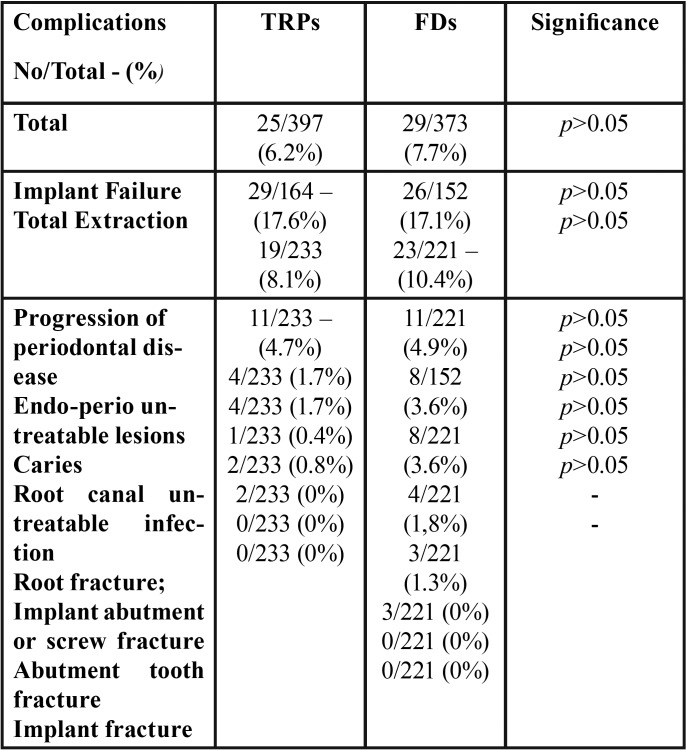
Major complications for TRPs group and FPs group.

MINOR COMPLICATIONS: ( Table 5 reports on the frequency of interventions carried out during the 15-year follow up for TRP and FD)

**Table 5:**
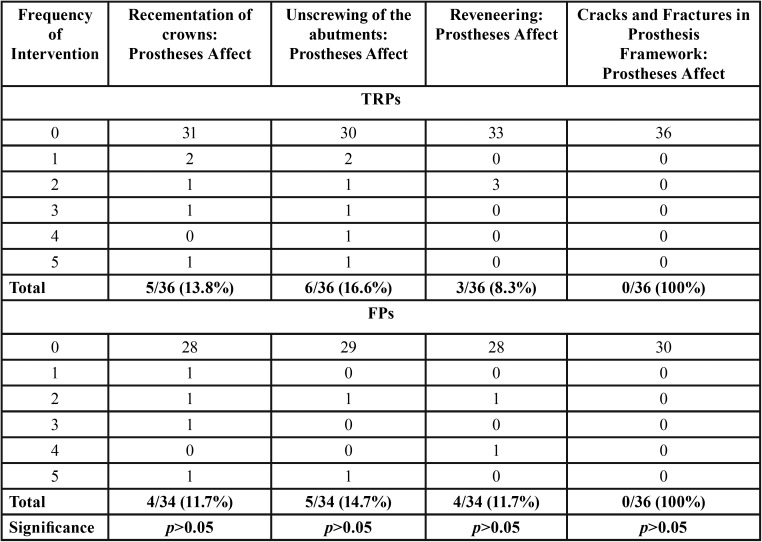
Minor complications for TRPs group and FPs group.

TRP group: Re-cementation of primary crowns was necessary for 5 of the 36 re-examined TRPs (13.8%). Sixty percent of these prostheses (3/5) required repeated re-cementations. With reference to the number of abutment teeth, 34 of the re-examined primary crowns (14.5%) were affected. Of these, 25.7% were repeatedly recemented. Re-screwing of abutment was necessary for 6 of the 36 re-examined TRPs (16.6%); 2 of 6 (33.3%) of these TRPs required repeated re-screwing. Re-veneering was necessary for 8.3% (3/36) of the prostheses that were re-evaluated, with 33.3% in need of multiple re-veneering. No cracks or fractures in the framework were observed in the TRPs.

FP group: Re-cementation of crowns was necessary for 4 of the 34 re-examined FDPs (11.4 %). Fifty percent of these prostheses (2/4) required repeated re-cementations. With reference to the number of abutment teeth, 26 of the re-examined 221 crowns (11.7%) were affected. Re-screwing of abutment was necessary for 5 of the 34 re-examined FPs (14.7 %); 1 of 2 (50 %) of these FPs required repeated re-screwing. Re-veneering was necessary for 11.7 % (4/34) of the prostheses that were re-evaluated. No cracks or fractures in the framework was observed in the FDPs

## Discussion

The choice to maintain and treat periodontally compromised teeth is related to the prognosis assigned to these teeth. Hopeless teeth have to be extracted as part of the initial (cause-related) therapy, whereas teeth with questionable prognosis that have not responded to the initial phase of periodontal therapy, and teeth with unpredictable prognosis that, after the active periodontal therapy (APT) still present a high risk, may have to be extracted (20-28). Remaining and properly treated teeth with healthy but markedly reduced periodontal support, are often mobile, and need to be splinted to enhance patient comfort (29). Once splinted, these teeth can carry extensive fixed prostheses for a very long time with survival rates of about 90%, provided the periodontal disease is eradicated and prevented from re-occurring (20-29). Several long-term follow-up studies (21,28) have shown that full arch fixed bridges can be placed and successfully maintained on a minimal number of abutment teeth with greatly reduced periodontal support, provided the prosthodontic treatment is preceded by adequate periodontal therapy, and followed by a support periodontal therapy (SPT).

The long-term high percentages of success in partially edentulous (healthy and periodontally compromised) subjects of osseointegrated implants (4,5) has considerably influenced the decision to maintain or to extract periodontitis-affected teeth. Today, there are opinions among some clinicians that the prognosis of complex, often time-consuming periodontal therapies may not match the advantage of implant therapy (6,7). Some researchers suggested that periodontally compromised teeth should be extracted for the sake of avoiding tooth-implant restorations, especially in full-arch prosthetic rehabilitation (32,33). On the other hand, several studies showed that periodontitis-susceptible patients are at higher risk for bone loss around implants (8-10). Such findings underscore the necessity of careful consideration regarding extracting periodontally compromised teeth, especially in patients requiring complex surgeries. Nevertheless, since full masticatory function requires a sufficient number of occluding posterior units to avoid cantilever effect (12), the use of supplemental implants could be recommended to enhance the clinical performance (34,35). The objectives of the current study were to assess the long term rates of abutment tooth loss, implant failure, and compare outcomes of full arch TRPs vs. full arch FPs supported by teeth-implants used to rehabilitate periodontally compromised dentitions after active periodontal therapy (ATP). Results showed that successful long-term retention of periodontally compromised abutment teeth with advanced bone loss is possible after APT if patients are enrolled in regular SPT. These outcomes are in agreement with other studies (20,28) indicating teeth retention for as long as possible, and to only replace them if absolutely necessary. To our knowledge, very few data have been published on using osseointegrated strategic implants associated with residual teeth in the rehabilitation of periodontally treated and maintained patients. Studies by Mitrani *et al.* (36) Hug *et al.* (37), Nickenig *et al.* (38), and Kaufman *et al.* (39) documented that the use of telescoping double crowns to combine implants and abutment teeth with RTP presents no negative effect on the prognosis of abutments teeth and implants. Furthermore, it has been suggested that in periodontally compromised patients, the presence of a frictional fit between the crown and coping sleeve may help prevent the intrusion also in teeth with reduced periodontal support (41). In the present study, using at least eight abutments per arch, full-arch FDs presented comparable long-term results of tooth loss and implant failure to full arch RTPs. The integration of several abutments might allow a favourable distribution of loading forces. Since abutment teeth with severely reduced but healthy periodontal tissue support still possess periodontal mechanoreceptors in the apical third of the root (41), the improved perception of teeth may help protect implants against occlusal overload. Compared to the RTP group, FDs group had a higher rate of caries and endodontic lesions. A rigid construction of a one-piece casting supported by teeth and implants reduces the retrievability of the prostheses, due to cement retention. To overcome this limitation (42), temporary cementation was used. Unlike temporary cemented FDs, TPRs allow for better retrievability.

A recent literature review (10) reported less favourable prognosis of dental implants installed in patients with a history of chronic periodontitis following successful periodontal therapy. On the other hand, outcomes of the current study showed that during the first 5 years of follow-up, the cumulative implant survival rate was 100%, but decreased between 6 and 15 years. This data indicates a higher susceptibility for implant failure after prolonged period of function. Similar results have been previously reported by Karoussis *et al.* (43) and Roccuzzo *et al.* (44), who speculated that it could be connected to multiple episodes of peri-implant infections. Both periodontitis and peri-implantitis have been proven to be associated with several host susceptible genes, such as interleukin-1, interleukin-6, tumor necrosis factor-alpha, and transforming growth factor beta (45,46). Therefore, patients with past or present periodontitis could be at greater risk of infection, peri-implantitis, bone loss, and eventually implant failure.

In each group of the current study, initial periodontal diagnosis, severity of the periodontal disease and smoking habits were identified as statistically significant factors in abutment tooth loss and implant failure.

At the end of the follow-up, in the TRP group, implant failure rate was 17.6%, while in the FP group, implant failure rate was 17.1 %. According to the collected data, it is possible to speculate that with the same starting conditions, implants in chronic periodontitis patients, treated with ATP and maintained in SPT, presented a lower longevity compared to abutment teeth. As far as the authors know, few studies evaluated the influence of periodontitis progression on onset and development of peri-implantitis in periodontally compromised and treated patients. Fardal & Linden (47) evaluated tooth loss and implant failure in patients refractory to periodontal treatment and reported that recurrence rather than history of disease increases risk of peri-implantitis. Results of the present study are in agreement with prior studies, since in each group, all patients who experienced abutment teeth loss presented implant failures too. On the contrary, only 2 subjects in TRP group and 1 subject in FP group experienced implant failure in the absence of periodontitis. Given the limited sample of the present study, conclusions cannot be drawn and further investigations are needed. However, the outcomes suggest that the retrievability of TRPs makes them the preferred option, allowing for peri-implant treatment and repair.

The present study lacks the random allocation of patients into treatment and control groups. Another limitation lies in the fact that the distribution and number of implants, abutment teeth, and pontics varied between the patients. However, to minimize the difference, considerable effort was taken to identify two groups which were as similar as possible regarding, age, gender, smoking, diagnosis (aggressive/chronic periodontitis), degree of bone loss, and number of teeth at the baseline.

## Conclusions

Within the limits of the present study, it is possible to conclude that in periodontally treated and maintained patients, 

• Full arch telescopic-retained retrievable prostheses, and full arch fixed prostheses, both supported by teeth-implants combination, have comparable and predictable long-term results if regular supportive periodontal therapy is implemented.

• Supplement, strategic implants, used in connection with residual abutment teeth, may enhance the clinical performance in unfavourable prosthetic baseline situations.

## References

[1] Nyman S, Lindhe J (1976). Persistent tooth hypermobility following completion of periodontal treatment. J Clin Periodontol.

[2] Nyman S, Lindhe J (1976). Prosthetic rehabilitation of patients with advanced periodontal disease. J Clin Periodontol.

[3] Nyman S, Lindhe J (1977). Considerations on the design of occlusion in prosthetic rehabilitation of patients with advanced periodontal disease. J Clin Periodontol.

[4] Graetz C, El-Sayed KF, Geiken A, Plaumann A, Sälzer S, Behrens E (2018). Effect of periodontitis history on implant success: a long term evaluation during supportive periodontal therapy in a university setting. Clin Oral Invest.

[5] Roccuzzo M, Bonino F, Aglietta M, Dalmasso P (2012). Ten-year results of a three arms prospective cohort study on implants in periodontally compromised patients. Part 2: clinical results. Clin Oral Implants Res.

[6] Alves CC, Correira AR, Neves M (2010). Immediate implants and immediate loading in periodontally compromised patients a 3-year prospective study. Int J Period Rest Dent.

[7] Donos N, Laurell L, Mardas N (2012). Hierarchical decisions on teeth vs. implants in the periodontitis-susceptible patient: the modern dilemma. Periodontol 2000.

[8] Karoussis IK, Salvi GE, Heitz-Mayfield LJA, Bragger U, Hammerle CHF, Lang NP (2003). Long-term implant prognosis in patients with and without a history of chronic periodontitis: a 10-year prospective cohort study of the ITIs Dental Implant System. Clin Oral Imp Res.

[9] Roccuzzo M, Bonino L, Dalmasso P, Aglietta M (2014). Long-term results of a three arms prospective cohort study on implants in periodontally compromised patients: 10-year data around sandblasted and acid-etched (SLA) surface. Clin Oral Imp Res.

[10] Souza V, Mardas N, Farias B, Petrie A, Needleman I, Spratt D (2016). A systematic review of implant outcomes in treated periodontitis patients. Clin Oral Imp Res.

[11] Lundgren D, Rylander H, Laurell L (2008). To save or to extract, that is the question. Natural teeth or dental implants in periodontitis susceptible patients: clinical decision making and treatment strategies exemplified with patient case presentations. Periodontology 2000.

[12] Hatch JP, Shinkai RS, Sakai S, Rugh JD, Paunovich ED (2001). Determinants of masticatory performance in dentate adults. Arch Oral Biol.

[13] Lang NP, Pjetursson BE, Tan K, Brägger U, Egger M, Zwahlen M (2004). A systematic review of the survival and complication rates of fixed partial dentures (FPDs) after an observation period of at least 5 years. II. Combined tooth-implant supported FPDs. Clin Oral Implants Res.

[14] Hoffmann O, Zafiropoulos GG (2012). Tooth-implant connection: a review. J Oral Implantol.

[15] Mamalis A, Markopoulou K, Kaloumenos K, Analitis A (2012). Splinting osseointegrated implants and natural teeth in partially edentulous patients: a systematic review of the literature. J Oral Implantol.

[16] Hita-Carrillo C, Hernández-Aliaga M, Calvo-Guirado JL (2010). Tooth-implant connection: a bibliographic review. Med Oral Patol Oral Cir Bucal.

[17] Schlumberger TL, Bowley JF, Maze GI (1998). Intrusion phenomenon in combination tooth-implant restorations: a review of the literature. J Prosthet Dent.

[18] Schei O, Waerhaug J, Lovdal A (1959). Alveolar bone loss as related to oral hygiene and age. J Periodontol.

[19] Hamp SE, Nyman S, Lindhe J (1975). Periodontal treatment of multirooted teeth. Results after 5 years. J Clin Periodontol.

[20] Nyman S, Lindhe J (1979). A longitudinal study of combined periodontal and prosthetic treatment of patients with advanced periodontal disease. J Periodontol.

[21] Graetz C, Schwendicke F, Kahl M, Dörfer CE, Sälzer S, Springer C (2013). Prosthetic rehabilitation of patients with history of moderate to severe periodontitis: a long-term evaluation. J Clin Periodontol.

[22] Lang NP, Tonetti MS (2003). Periodontal risk assessment (PRA) for patients in supportive periodontal therapy (SPT). Oral Health Prev Dent.

[23] Eickholz P, Kaltschmitt J, Berbig J, Reitmeir P, Pretzl B (2008). Tooth loss after active periodontal therapy. 1: patient-related factors for risk, prognosis, and quality of outcome. J Clin Periodontol.

[24] Chambrone L, Chambrone D, Lima LA, Chambrone LA (2010). Predictors of tooth loss during long-term periodontal maintenance: a systematic review of observational studies. J Clin Periodontol.

[25] Baumer A, El Sayed N, Kim TS, Reitmeir P, Eickholz P, Pretzl B (2011). Patient related risk factors for tooth loss in aggressive periodontitis after active periodontal therapy. J Clin Periodontol.

[26] Baumer A, Pretzl B, Cosgarea R, Kim TS, Reitmeir P, Eickholz P (2011). Tooth loss in aggressive periodontitis after active periodontal therapy: patient-related and tooth-related prognostic factors. J Clin Periodontol.

[27] Meyer-Baumer A, Pritsch M, Cosgarea R, El Sayed N, Kim TS, Eickholz P (2012). Prognostic value of the periodontal risk assessment in patients with aggressive periodontitis. J Clin Periodontol.

[28] Yi SW, Ericsson I, Carlsson GE, Wennström JL (1995). Long-term follow-up of cross-arch fixed partial dentures in patients with advanced periodontal destruction. Acta Odontol Scand.

[29] Lindhe J, Nyman S (1977). The role of occlusion in periodontal disease and the biological rationale for splinting in treatment of periodontitis. Oral Sci Rev.

[30] Nyman S, Ericsson I (1982). The capacity of reduced periodontal tissues to support fixed bridgework. J Clin Periodontol.

[31] Muddugangadhar BC, Amarnath GS, Sonika R, Chheda PS, Garg A (2015). Meta-analysis of failure and survival rate of implant supported single crowns, fixed partial denture and implant tooth supported prostheses.

[32] Cordaro L, Ercoli C, Rossini C, Torsello F, Feng C (2005). Retrospective evaluation of complete-arch fixed partial dentures connecting teeth and implant abutments in patients with normal and reduced periodontal support. J Prost Den.

[33] Tsaousoglou P, Michalakis K, Kang K, Weber H, Sculean A (2017). The effect of rigid and non-rigid connections between implants and teeth on biological and technical complications: a systematic review and a meta-analysis. Clin Oral Imp Res.

[34] Krennmair G, Krainhöfner M, Waldenberger O, Piehslinger E (2007). Dental implants as strategic supplementary abutments for implant-tooth-supported telescopic crown-retained maxillary dentures: a retrospective follow-up study for up to 9 years. Int J Prosthodont.

[35] Rinke S, Ziebolz D, Ratka-Krüger P, Frisch E (2015). Clinical Outcome of Double Crown-Retained Mandibular Removable Dentures Supported by a Combination of Residual Teeth and Strategic Implants. J Prosthodont.

[36] Mitrani R, Brudvik JS, Phillips KM (2003). Posterior implants for distal extension removable prostheses: a retrospective study. Int J Periodontics Restorative Dent.

[37] Hug S, Mantokoudis D, Mericske-Stern R (2006). Clinical evaluation of 3 overdenture concepts with tooth roots and implants: 2-year results. Int J Prosth.

[38] Nickenig HJ, Spiekermann H, Wichmann M, Andreas SK, Eitner S (2008). Survival and complication rates of combined tooth-implant supported fixed and removable partial dentures. Int J Prosth.

[39] Kaufmann R, Friedli M, Hug S, Mericske- Stern R (2009). Removable dentures with implant support in strategic positions for up to 8 years. Int J Prosth.

[40] Guarnieri R, Ippoliti S (2018). Restoration of Periodontally Compromised Dentitions Using Telescopic Full-Arch Retrievable Prosthesis Supported by Tooth-Implant Combination: A Long-Term Retrospective Study. Int J Periodontics Restorative Dent.

[41] Nyman S, Lindhe J, Lundgren D (1975). The role of occlusion for the stability of fixed bridges in patients with reduced periodontal tissue support. J Clin Periodontol.

[42] Borg P, Puryer J, McNally L, O'Sullivan D (2016). The Overall Survival, Complication-Free Survival, and Related Complications of Combined Tooth-Implant Fixed Partial Dentures: A Literature Review. Dent J.

[43] Karoussis IK, Salvi GE, Heitz-Mayfield LJA, Bragger U, Hammerle CHF, Lang NP (2003). Long-term implant prognosis in patients with and without a history of chronic periodontitis: a 10-year prospective cohort study of the ITIs Dental Implant System. Clin Oral Impl Res.

[44] Roccuzzo M, Bonino L, Dalmasso P, Aglietta M (2014). Long-term results of a three arms prospective cohort study on implants in periodontally compromised patients: 10-year data around sandblasted and acid-etched (SLA) surface. Clin Oral Impl Res.

[45] Laine ML, Crielaard W, Loos BG (2012). Genetic susceptibility to periodontitis. Periodontology 2000.

[46] Ghassib I, Chen Z, Zhu J, Wang HL (2019). Use of IL-1 β, IL-6, TNF-α, and MMP-8 biomarkers to distinguish peri-implant diseases: A systematic review and meta-analysis. Clin Impl Dent and Rel Res.

[47] Fardal O, Linden GJ (2008). Tooth loss and implant outcomes in patients refractory to treatment in a periodontal practice. J Clin Periodontol.

